# Open-Source Photochemistry in the Organic Chemistry Teaching Laboratory

**DOI:** 10.1021/acsomega.5c06916

**Published:** 2025-09-30

**Authors:** K. C. Levandoski, Christopher Jiotis, James A. Kenar, Steven J. Kregel, Shawn D. Montag

**Affiliations:** Mund-Lagowski Department of Chemistry and Biochemistry, 6496 Bradley University, Peoria, Illinois 61625, United States

## Abstract

We report the development of an undergraduate organic chemistry laboratory exercise that utilizes a set of low-cost, open-source photoreactors to introduce students to photochemical principles. A series of nine 3D printed photoreactors were constructed with emission maxima centered from the near-UV to the near-IR. Students used these photoreactors to measure the impact of photon wavelength on the bromination of bibenzyl. The class data was pooled, and the students rationalized the observed wavelength dependence of the reaction. Results from a postlab survey indicated that students believed this laboratory activity increased their understanding of photochemistry, their ability to formulate their own hypotheses, and their ability to adapt and learn from their mistakes.

## Graphical abstract

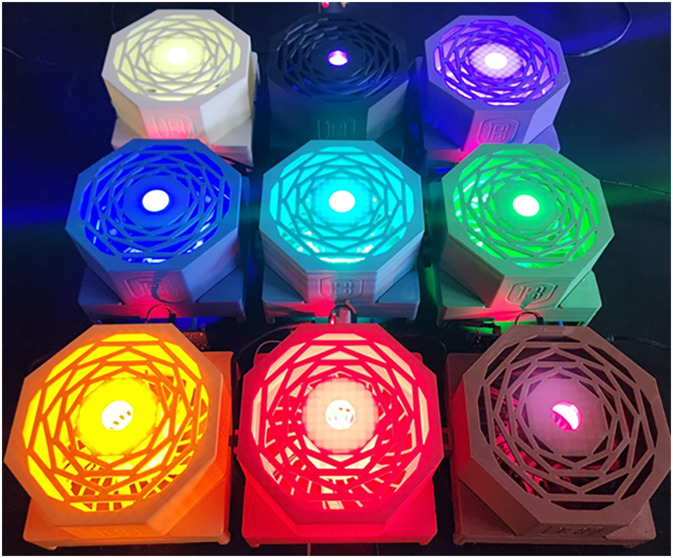

## Introduction

The role of photochemistry in modern organic synthesis continues to grow.^1–5^ Photoinduced reactions have been utilized to drive a myriad of chemical transformations, including photoredox catalysis,^6,7^ polymerization reactions,^8^ and free radical reactions.^9^ Additionally, photochemistry is becoming more widely adopted throughout the chemical industry, from pharmaceutical development to waste remediation.^10,11^ Modern photochemistry experiments are also moving away from power-hungry broadband lamps and toward high-efficiency single-color LED light sources that offer higher efficiency, longer life, and lower cost.^12–14^ Thus, it is important that undergraduate students be exposed to and educated about the capabilities and considerations of using photochemistry in organic labs.^15–21^

With this in mind, we sought to modify a well-established free radical bromination reaction of 1,2-diphenylethane (bibenzyl) that has been performed at many universities using a variety of traditional methods and conditions.^22–24^ The bromination of bibenzyl is a convenient reaction for the undergraduate laboratory due to the formation of a solid, filterable product. The reaction also produces only one diastereomer, rather than a racemic mixture. However, radical substitution with bromine is usually quite time-consuming, and reaction procedures have mostly called for the use of carbon tetrachloride as a solvent, which is undesirable due to its harmful effects.^25^ For these reasons, we chose to modernize this familiar experiment to utilize LED-driven photochemistry and teach students about the importance of the wavelength on the rate of photochemical reactions.

## Overview of the Laboratory Experiment

The experiment described herein was used to introduce students to the fundamentals of photochemistry. In this case, students reacted bibenzyl with a solution of bromine in dichloromethane under irradiation from an LED photoreactor to obtain *meso*-1,2-dibromo-1,2-diphenylethane, as shown in Scheme 1.

**Scheme 1:**
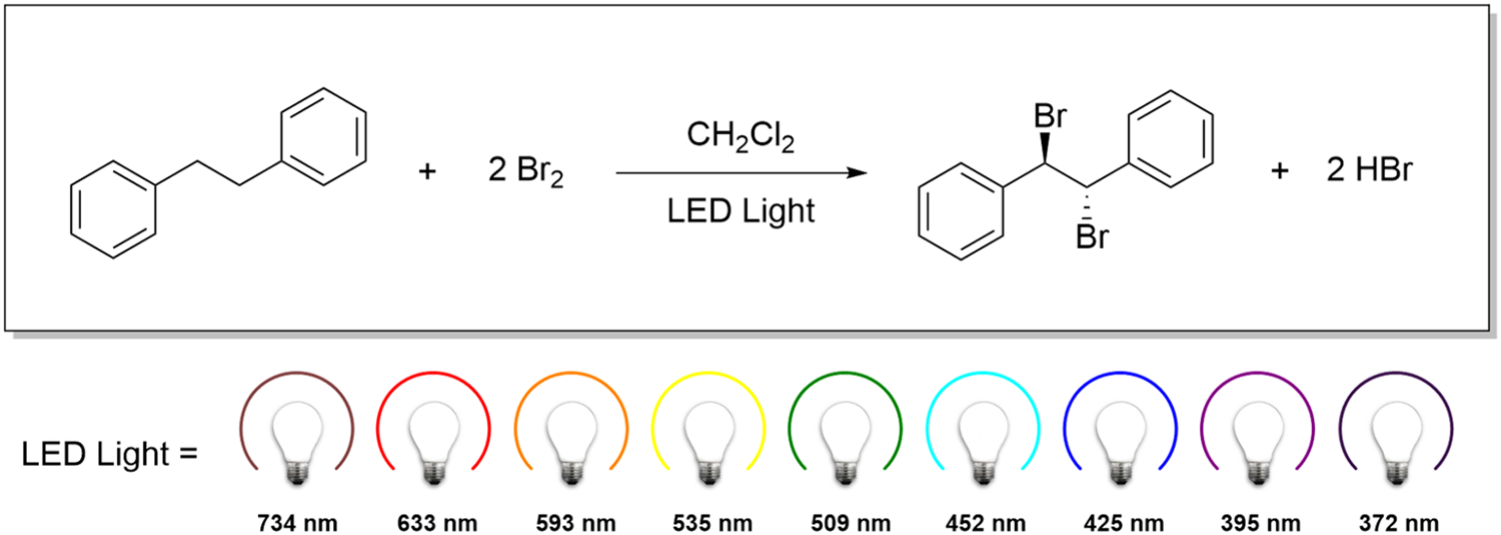
Bromination of Bibenzyl Using Various Wavelengths Produced by LED Photoreactors

The students analyzed the purified product using melting point analysis, infrared (IR) spectroscopy, and nuclear magnetic resonance (NMR) spectroscopy. This 2-week (6 h) experiment was tested for 2 years by over 12 different sections, each containing 12–14 students in the organic chemistry laboratory. Before the first week of the lab, students are required to look up safety information regarding all the starting materials and solvents to be used in the lab. Students are given the absorbance spectrum for bromine in dichloromethane and the emission spectrum for each LED photoreactor that they will use in the lab. These spectra are shown in Figure 1A, and the students use them to develop a hypothesis before starting the lab. The laboratory discussion involves a background on LEDs, the advantages of LEDs over conventional lighting counterparts, and the use of light to perform organic chemistry reactions.

**Figure 1:**
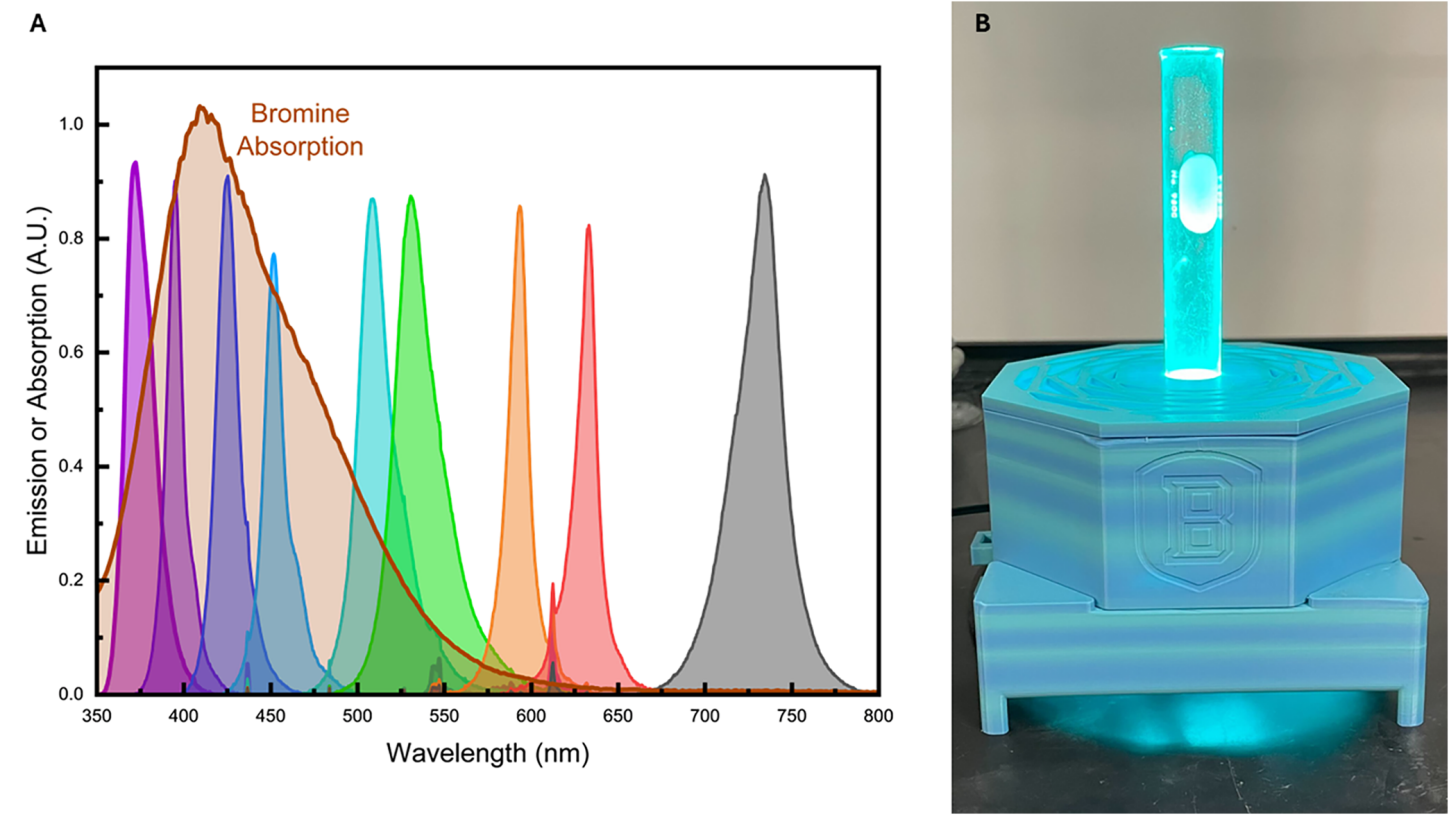
(A) Absorbance spectrum for bromine in dichloromethane and emission spectrum for each LED photoreactor. (B) 509 nm photoreactor with a test tube.

The students are assigned in groups of two and given an LED photoreactor that emits a band of light centered on a specific wavelength. In our experiment, these band centers are one of the following: 372, 395, 425, 452, 509, 535, 593, 633, or 734 nm. The reaction times for the bromination reaction are highly dependent on the wavelength of light used by the student. Students are then expected to run a test reaction with their assigned photoreactor and observe for a color change. The LED is turned on, and a stopwatch is started. As the reaction progresses, the dark red solution starts to bubble and turn colorless over time, indicating Br_2_ consumption and the production of HBr gas. A sealed reaction vessel would better contain the volatile and corrosive Br_2_ vapor, but due to the production of gaseous HBr, we use an open-topped reaction vessel to prevent pressure buildup. After the reaction solution becomes colorless and the appearance of a white, crystalline solid is observed, the total reaction time is noted, and any remaining bromine is quenched with cyclohexene. The product, once thoroughly dried, is analyzed using IR, NMR, and melting point analyses.

In the second week of the experiment, students are allowed to run multiple reaction trials (typically 5 to 10) using their designated LED photoreactor. The experimental setup using the 509 nm photoreactor is shown in Figure 1B for reference. By running the experiment multiple times, students can reinforce their overall laboratory skills, fix any previous mistakes, and minimize errors associated with their observed reaction times (sometimes it can be difficult to determine when the solution is colorless; for example, the bromine solution is red and one of the LEDs emits red light). The data from each LED photoreactor is tabulated on the whiteboard, and students are responsible for calculating the average and standard deviation of the reaction time for each photoreactor. The results are then graphed. After the lab is completed and the data is collected, students are then required to complete a worksheet and confirm or reject their initial hypothesis (the worksheet is provided in the Supporting Information).

## Photoreactor Construction and Characterization

The photoreactors used in this project are modified versions of the previously reported Wisconsin Photoreactor Platform (WPP).^26^ The WPP is an open-source, 3-D printed, LED-based photoreactor designed for ease of use and custom modifications. Full assembly instructions for the WPP are available on GitHub.^27^ Our modifications to the original design and considerations for use by undergraduates are described below. The base of the WPP holds an industry-standard high-powered LED chip mounted to an aluminum heat sink and cooled by a computer fan. The intensity of the LEDs within each photoreactor was controlled by utilizing the analog driver board included in the WPP designs. Electrical components for the analog driver board were ordered from Mouser.com, and empty printed circuit boards were ordered from OSHPark. The circuit boards were assembled by an undergraduate student as part of an independent research project at Bradley University. This student, with no previous experience in assembling electronics, was able to assemble the circuit boards for our nine photoreactors in about 40 h.

The bases and reaction chamber housings for our photoreactors were 3-D printed from poly­(lactic acid) (PLA) on a Bambu Lab X1-Carbon 3-D printer. We chose to use PLA as it is one of the most common 3-D printing materials and is compatible with a wide range of entry-level printers. Our printer has a 254 × 254 mm print bed and can produce an entire photoreactor system in a single print; however, smaller printers should be capable of printing the WPP piecewise, as long as the print bed is at least 140 × 140 mm. Each photoreactor required ∼200 g of PLA to produce, which roughly corresponds to a cost of around 4 dollars, depending on the PLA filament source. As the WPP was originally designed for use in a research lab, the previously published reaction chamber housings were all designed to enable the simultaneous irradiation of multiple reaction vessels. In this experiment, we wanted the students to focus on the time required for a single reaction, and thus, we designed our own reaction chamber housing using Autodesk Inventor (free license for academic use available from Autodesk). Our custom reaction chamber holds a single test tube directly above the high-powered LED and contains nearly all of the stray light, greatly reducing the impact of the photoreactor on the surrounding lab space. Including the cost of the 3-D printer filament, the total cost for a single photoreactor is under 100 dollars, as reported in the original WPP publication.^26^ All of the modified photoreactor fabrication files used in this work are available in the Supporting Information.

As part of their laboratory exercise, students are given the emission spectra from the 9 photoreactors that utilize different colored LEDs. The emission spectra were acquired using an OceanInsight OceanHDX spectrometer with a fiber-coupled probe. To avoid saturating the spectrometer, the emission spectra were acquired at the minimum power output achievable with the WPP driver board. All emission spectra were acquired at the same power level, and thus the intensities shown in Figure 1 are representative of the relative luminous intensities of each photoreactor.

## Experimental Protocol

In the first 3 h of the laboratory session, each student group is assigned one photoreactor with a specific wavelength. The students dissolve 1.0 mmol of bibenzyl in 1 mL of dichloromethane in a test tube. Once the bibenzyl is dissolved, 6 mL of a 53 mg/mL concentration of bromine solution in dichloromethane (∼2.0 mmol bromine) is added to the test tube via buret. A stir bar is also added, and then the test tube is placed in the photoreactor. The photoreactor is placed on a stir plate, and once stirring is started, the photoreactor is turned on. The students start timing the reaction once they switch on the photoreactor. When the reaction turns colorless and the reaction is completed, the timer is stopped, and one drop of cyclohexene is added to the colorless solution to quench any remaining bromine. The test tube is placed in an ice bath for ten minutes. The reaction mixture is then filtered via Hirsch filtration to obtain a solid white product. The students dry their *meso*-stilbene dibromide product and obtain the mass and melting point. An IR spectrum of the product is obtained using a Nicolet iS50R FT-IR with an iS50 ATR attachment, and an NMR spectrum is obtained using a Jeol 400 MHz spectrometer.

The following week, each student replicates this experiment multiple times (5–10, depending on the light source and time constraints). Note: The students do not have to determine the melting point, IR, and NMR of the product every time, but it is recommended to determine the mass and have students see whether the percent yield stays constant. The students write their reaction times for each run on the whiteboard, use the compiled class data to create graphs of their reaction times with respect to the LED wavelength, and answer the worksheet questions. The full experimental details are provided in the Supporting Information.

## Hazards

Eye protection and proper gloves should be worn at all times during the experiment, and the work should be performed in a fume hood. For student groups using the 372 and 395 nm photoreactors, special UV protective goggles were used. Bromine is corrosive, toxic, and can cause serious irritation or burns to the skin, eyes, and lungs; therefore, it should only be handled in a fume hood with proper lab goggles and gloves. To minimize the chances of student contact, the bromine solution is added to a buret by the instructor. Bibenzyl may cause skin irritation. Cyclohexene is a skin and eye irritant, should not be inhaled, and should be stored in a well-ventilated area. To minimize student exposure to hazardous chemicals, the instructor goes to each fume hood and quenches any remaining bromine for the students. Dichloromethane can cause skin and eye irritation and is a suspected carcinogen. It is worth noting that due to the recent EPA requirements regarding the use of dichloromethane, we explored a range of potential solvents for this reaction, but none were as effective as dichloromethane. Deuterochloroform causes skin and respiratory irritation, may cause chemical burns, and is suspected to be carcinogenic. All halogenated waste is disposed of in a waste container labeled “halogenated organic waste,” while all other waste is disposed of in a waste container labeled “non-halogenated organic waste.”

## Student Results and Discussion

The bromination reaction was used to probe the effects of photon wavelength on the reaction between bibenzyl and bromine. The percent yield for this specific reaction did not appear to be heavily influenced by the wavelength of light used, as long as the reaction was allowed to proceed until the red color of the bromine dissipated. Table 1 shows the student data for the average percent yield and melting point of the *meso*-stilbene dibromide product at each specific wavelength.

**Table 1:** LED Wavelength vs. Average Percent Yield

**LED wavelength** (nm)	**percent yield averaged** (%)	**melting point** (°C)
372	54	234–238
395	57	232–239
425	61	235–238
452	62	235–240
509	58	234–238
535	53	234–238
593	55	235–238
633	53	236–239
734	44	239–240

Overall, the percent yields varied from 0% to 74% for this reaction. Very few students attained a 0% yield for this reaction, and it was discovered that for those students, the test tubes that they had used had been cleaned with acetone and not allowed to dry in the oven before setting up the reaction, resulting in the quenching of bromine. The average percent yield for the reactions run with each LED was approximately 50–60%, with the only exception being the reactions run with the infrared LED photoreactor (734 nm). Bromine’s low absorption in this region indicates that these reactions may have been thermally, rather than photolytically, initiated. Students were also responsible for running and analyzing the IR and ^1^H NMR spectra of their products (experimental spectra provided in the Supporting Information).

The reaction times from the student data for the bromination of bibenzyl using LEDs of specific wavelengths followed the expected trend, as shown in Figure 2.

**Figure 2:**
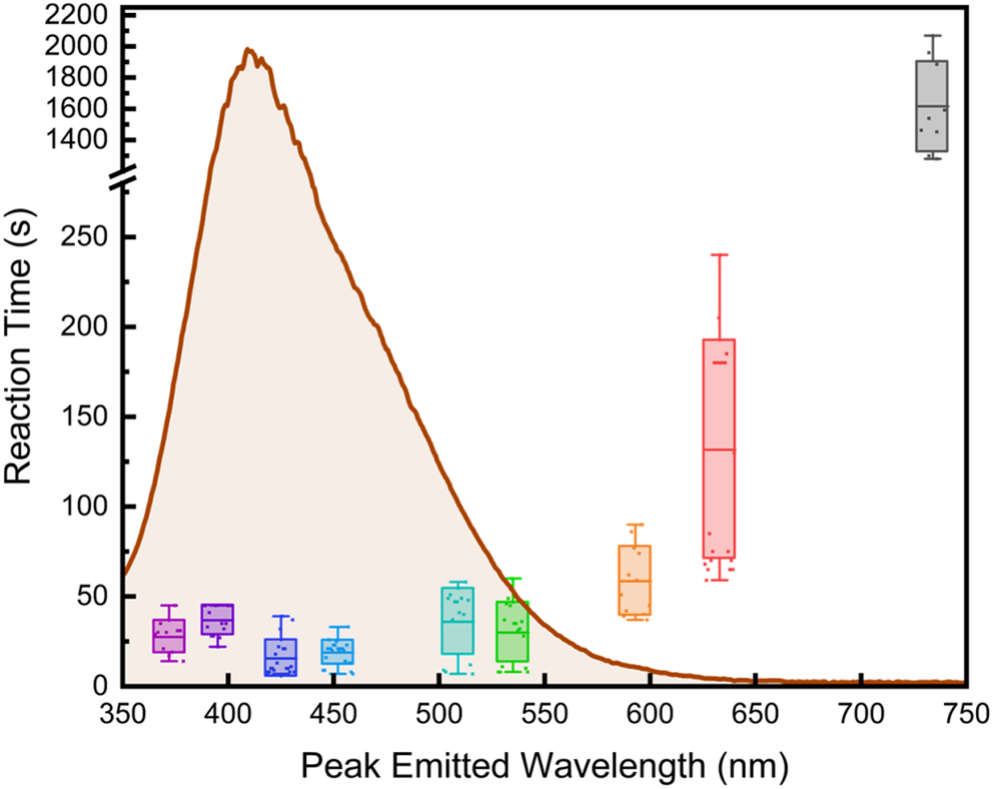
Bromination of bibenzyl using LED photoreactors of specific wavelengths with respect to the reaction time averages.

Bromine has been shown to have a maximum absorption at around 405 nm; therefore, it is hypothesized that 395 and 425 nm light would produce the fastest reaction time. Statistically, the data show minimal difference between reaction times using 372, 395, 425, and 452 nm light, with the average reaction time being between 20–40 s. It is worth noting that because of the short reaction times, a small amount of error, such as not setting the stopwatch immediately upon starting the photoreactor to initiate the reaction, can cause a drastic difference in the reaction time (i.e., 10 vs 12 s is a large difference at shorter reaction times, so students run 5–10 reactions and average the reaction times). At 509 and 535 nm, the reaction tends to take a little longer, but the reaction time consistently stays under 60 s for the students’ experiments. The 593 nm photoreactor was able to perform the bromination reaction consistently between 60 and 90 s. The student data from the 633 nm light had the widest range of reaction times, ranging from 80–190 s, with an average reaction time of around 140 s. Even though the reaction time for the 633 nm light has the widest range, it still follows the correct trend, given that bromine does not absorb light effectively in that region. Lastly, the bromination reaction using the 734 nm photoreactor was possible; however, it took 1600 s (∼26.5 min) on average, importantly demonstrating that the bromination reaction drastically slows down where bromine does not absorb light well. Overall, the students demonstrated their ability to obtain and interpret experimental data from the bromination reaction and validate the hypothesized dependence of the reaction rate on the specific wavelength of light used.

## Discussion on the Effectiveness of LED Bromination Methodology on Pedagogical Goals

The bromination of bibenzyl using LED photoreactors in the undergraduate organic chemistry laboratory provided an opportunity for students to gain important skills, in line with the pedagogical goals of this work. The main goals associated with this work were that students would be able to

- Hypothesize the rate of the bromination reaction at different wavelengths, given the absorption spectrum of bromine.
- Utilize noncommercial equipment to investigate specific scientific goals.
- Analyze ^1^H NMR and IR spectra.
- Calculate the percent yield.
- Understand the sources of error and variability.
- Promote mechanistic thinking and link practical results to chemical principles.
- Perform organic laboratory techniques with precision and safety.

Upon completion of the laboratory experiment, students were able to complete a worksheet related to the main objectives of the experiment (worksheet provided in the Supporting Information). An analysis of student responses to the worksheet questions showed that there was an overwhelmingly positive impact on the effectiveness of teaching students about reaction rates and LED lights. Students were able to successfully calculate the minimum wavelength of light with sufficient energy required to break the Br–Br bond, analyze ^1^H NMR and IR spectra, and calculate the cost of performing each reaction at each wavelength, given their average reaction times.

Each student who performed the experiment was surveyed afterward and asked questions related to their learning and the laboratory experiment. The survey was a Likert-style survey with values ranging from 1 to 5, with 1 indicating a strong disagreement and 5 indicating a strong agreement. The prompts and averages are reported in Table 2.

**Table 2:** Survey on Student Learning

in this laboratory experiment, I···	average score
formulated and tested my own hypothesis	4.45
used IR and ^1^H NMR to effectively analyze my product	3.89
tried to reason through potential sources of error	4.67
practiced safe handling of chemicals	4.81
adapted in response to any mistakes made in the trial run	4.34
was excited to do chemistry	4.72
considered whether my data made sense	4.60

Most students agreed that this laboratory experiment provided them with a meaningful opportunity to formulate and test their hypotheses. They also reported that they were able to adapt in response to any mistakes that they made in the trial run, a valuable outcome given that the lab was intentionally designed to allow multiple reaction trials to let students make mistakes and try again. Notably, the highest average score was for practicing safe handling of chemicals, which is particularly encouraging because it was emphasized during the prelab that dichloromethane and bromine are unsafe chemicals, especially when handled improperly. An equally compelling result was the strong agreement that this experiment excited students to do chemistry, which is important because their engagement and enthusiasm in the laboratory can promote a deeper conceptual understanding. The lowest reported average was for using IR and ^1^H NMR to effectively analyze their products. While expected, since students were just beginning to build confidence with ^1^H NMR at this stage in the curriculum, it was reassuring to see that students still expressed agreement with this statement, indicating a growing comfort level with spectral analysis.

## Conclusions

A 2-week free radical bromination of bibenzyl reaction utilizing bromine and low-cost, open-source LED photoreactors for the organic chemistry laboratory was successfully implemented. The reaction was easy for students to set up, and the reaction rates for the LED photoreactors allowed for multiple iterations of the experiment, allowing the students to correct for earlier mistakes and attain reliable and repeatable results. The students were able to combine the data with their classmates to gain a full picture of how each specific wavelength affected the reaction time. The product from the reaction underwent no further purification and produced high-quality ^1^H NMR and IR spectra that were easily analyzed.

## Supplementary Material



## Notes

Detailed instructions for students, instructor notes, NMR spectra, and modified WPP fabrication files (ZIP)
