# Supplementary material for: Open-Source Photochemistry in the Organic Chemistry Teaching Laboratory
Source: ACS Omega. 2025 Sep 30;10(40):47415–20. doi: 10.1021/acsomega.5c06916 (PMC12529206; doi:10.1021/acsomega.5c06916)
Supplement: Supplementary file 1 [file ao5c06916_si_001.zip › Supporting Information for LED Bromination.docx]

**Supporting Information**

Open-Source Photochemistry in the Organic Chemistry Teaching Laboratory

*K. C. Levandoski, Christopher Jiotis, James A. Kenar, Steven J. Kregel,* and Shawn D. Montag**

Mund-Lagowski Department of Chemistry and Biochemistry, Bradley University, Peoria, Illinois 61625, United States

Email: [smontag@fsmail.bradley.edu](mailto:smontag@fsmail.bradley.edu)

Email: sjkregel@fsmail.bradley.edu

1. **Student Instructions**
   1. **Light Induced Free Radical Bromination of Bibenzyl**
   2. **Mechanism**
   3. **Before coming to Lab**
   4. **Procedure**
   5. **Required Materials**
   6. **Hazards Table**
   7. **Disposal of Waste**
   8. **Chemical Hazards**
2. **Notes for instructors**
   1. **Table of Chemicals Filled Out**
   2. **Additional Spectra**
3. **LED Bromination Lab Worksheet**
4. **Student Instructions**

Light Induced Free Radical Bromination of Bibenzyl

**Objective:** During this activity students will investigate the influence of different color LED lights to induce a radical substitution reaction that occurs between bromine and benzylic hydrogens. Students will monitor the reaction as a function of time and determine the identity of the isolated product (both the regiochemistry and the stereochemistry).

**Background:** As shown in **Figure S1**, free radical bromination of 1,2-diphenylethane (also known as bibenzyl) (**1**), using two equivalents of bromine, yields predominantly *meso-*1,2-dibromo-1,2-diphenylethane (stilbene dibromide) (**2).**

Halogens (fluorine, chlorine, bromine or iodine) can undergo bond cleavage (homolysis) when exposed to energy (heat or light). In the homolytic cleavage, each bromine atom gets one electron from the bond they share. After cleavage each bromine atom possesses seven electrons and exists as radical. The reactivity of the halogens goes as the following: F2 > Cl2 > Br2 > I2.

In this reaction, light serves as the energy source and is used to induce the homolytic cleavage of the Br-Br bond. Published work has indicated that bromine has a bond dissociation energy, BDE = 194 kJ/mol. This represents the minimum amount of energy required to homolytically cleave the Br-Br bond. The resulting bromine radical is well known and can abstract hydrogen atoms from C-H bonds to form nucleophilic carbon radicals

Light emitting diodes (LEDs) will be used as the light source. LEDs are solid-state semiconductor devices that emit light directly without using a bulb. LEDs can emit light across the visible light (**Figure S2**) range with discrete, narrow excitation peaks. The intensity of LEDs can be precisely controlled in 1% increments. LED light sources have a long lifetime and their brightness does not decrease with time. The long lifetime and stability of LEDs mean they are excellent for experiments running across many days or weeks. LEDs have a high upfront cost. However, they are often more economical due to their low maintenance costs, long lifetime and stability than traditional high-powered lamps. Maintenance costs are reduced as they do not need to be aligned or disposed of as hazardous waste. LEDs are more energy efficient than other light sources, saving money on electricity.

Source [Fluorescence light sources: A comparative guide (scientifica.uk.com)](https://www.scientifica.uk.com/learning-zone/choosing-the-best-light-source-for-your-experiment)

Figure S1: Proposed First Radical Bromination Steps of Bibenzyl


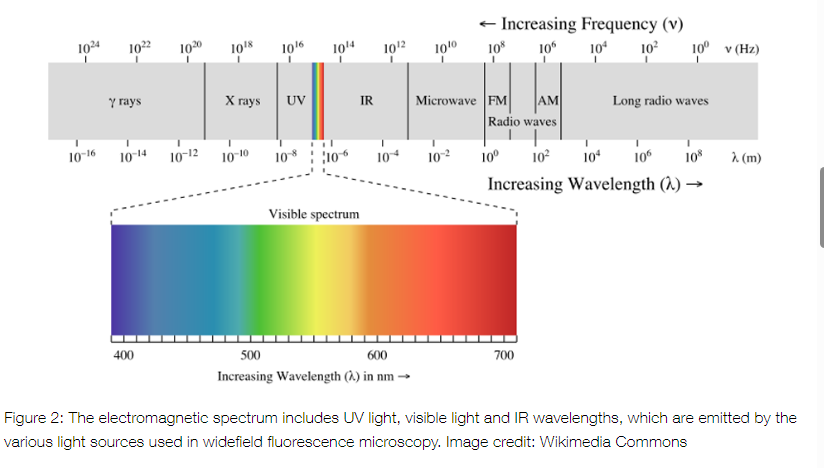


Figure S2: The electromagnetic spectrum includes UV light, visible light, and IR wavelengths, which are emitted by various light sources used in widefield fluorescence microscopy. Image credit: Wikimedia Commons.

*Meso-*1,2-dibromo-1,2-diphenylethane is the major product for several reasons:

1) Alkyl radicals are more stable than aryl radicals, and therefore bromine atoms abstract hydrogens from sp3 hybridized carbons rather than sp2 hybridized carbons.

2) Bromine radicals seek hydrogens of higher electron density and therefore remove a hydrogen from a carbon without a bromine already attached. (Bromine radicals are often referred to as electrophilic radicals.)

3) 1,2-dibromo-1,2-diphenylethane has low solubility in dichloromethane, the reaction solvent, and therefore it precipitates out of solution as it forms. In addition to being a regioselective reaction, this particular reaction is stereoselective. The stereoselectivity of this reaction can be understood by analyzing the transition state for the mechanistic step that determines stereochemistry. Below are Newman projections of the possible transition states for this mechanistic step.

**Prior to the start of the first week of lab:**

1. Be sure to have filled out your lab notebook with all required pre-laboratory information, including purpose, table of physical constants, chemical safety, reaction scheme, etc.

**PROCEDURE:**

**Week 1:**

***CAUTION:*** *Perform all operations in a* ***HOOD****. Do not breathe the vapors of bromine, dichloromethane, or hydrogen bromide. Immediately report any bromine spill to your instructor. (Instructors: Use sodium thiosulfate solution to wash bromine spills.)*

**The Reaction**

1. Place 1.0 mmol of 1,2-diphenylethane in a clean, dry, 6” test tube and add 1 mL of dichloromethane to dissolve the solid.
2. Calculate the amount of the bromine solution needed use 2.0 equivalents of bromine. The concentration of this solution is 53 mg/mL in dichloromethane) and add this amount to the test tube via the buret found in the prep hood.
3. Begin timing your reaction once you illuminate the resulting solution with your chosen light (either infrared, red, orange, yellow, cyan, green, blue, violet, or UV) until the solution changes from red to colorless. The bromination should proceed with the evolution of HBr and the formation of the solid product. Once colorless, stop your reaction timer and cool the test tube in an ice-water bath in the **HOOD**. Write your reaction time and color on the board.
4. Before removing the test tube from the hood, the instructor will add 1 or 2 drops of cyclohexene to react with any excess bromine.
5. Isolate the solid product by Hirsch filtration (**PREWEIGH** the filter paper) and wash the material with two 1 mL portions of cold *tert-*butyl methyl ether. Pull air through the product for at least 10 minutes to thoroughly dry the dibromide.
6. Calculate % yield of your product.
7. Obtain the melting point of your product.
8. Run NMR and IR on your product as well.

**Clean Up:**

1. Pour the filtrate from the vacuum filtration into the "used chemicals" container in the hood.
2. Thoroughly wash the filter flask, Hirsch funnel, and test tube using soap and water, rinse with DI water, then acetone and finally air dry.

Note: m.p. *meso*-1,2-dibromo-1,2-diphenylethane: 241 oC dec.

m.p. *R,R/S,S*-1,2-dibromo-1,2-diphenylethane: 114 oC

**Week 2:**

***CAUTION:*** *Perform all operations in a* ***HOOD****. Do not breathe the vapors of bromine, dichloromethane, or hydrogen bromide. Immediately report any bromine spill to your instructor. (Instructors: Use sodium thiosulfate solution to wash bromine spills.)*

1. Place 1.0 mmol of 1,2-diphenylethane in a clean, dry, 6” test tube and add 1 mL of dichloromethane to dissolve the solid.
2. Calculate the amount of the bromine solution needed use 2.0 equivalents of bromine. The concentration of this solution is 53 mg/mL in dichloromethane) and add this amount to the test tube via a buret.
3. Begin timing your reaction once you illuminate the resulting solution with your chosen light (either infrared, red, orange, yellow, cyan, green, blue, violet, or UV) until the solution changes from red to colorless. The bromination should proceed with the evolution of HBr and the formation of the solid product. Once colorless, stop your reaction timer and cool the test tube in an ice-water bath in the **HOOD**. Write your reaction time and color on the board.
4. Before removing the test tube from the hood, add 1 or 2 drops of cyclohexene to react with any excess bromine.
5. Repeat the reaction 5 to 10 times (as time in lab allows) and write all reaction times on the board. Note: Infrared will take the longest and likely will only get 1 run.

**Clean Up:**

1. Pour the filtrate from the vacuum filtration into the "used chemicals" container in the hood.
2. Thoroughly wash the filter flask, Hirsch funnel, and test tube using soap and water, rinse with DI water, then acetone and finally air dry.

Weigh the product and determine its melting point and percent yield of the reaction.

Note: m.p. *meso*-1,2-dibromo-1,2-diphenylethane: 241 oC dec.

m.p. *R,R/S,S*-1,2-dibromo-1,2-diphenylethane: 114 oC

**Required Materials:**

Bromine

Dichloromethane

1,2-diphenylethane (bibenzyl)

Cyclohexene

*Tert*-butyl methyl ether

CDCl3

**Hazards Table:**

Please look up the physical properties and hazards for each chemical being used in this research experiment and fill out the following table in your laboratory notebook. Note: You can list hazards below table if they don’t fit well.

Table S1. Table of Physical Properties and Chemical Hazards

| Substance | Formula | MM (g/mol) | Density  (g/ml) | M.P.  (°C) | B.P.  (°C) | Amount needed (mols) | Hazards |
| --- | --- | --- | --- | --- | --- | --- | --- |
| Bromine |  |  |  |  |  |  |  |
| CH2Cl2 |  |  |  |  |  |  |  |
| 1,2-diphenylethane |  |  |  |  |  |  |  |
| *Cyclohexene* |  |  |  |  |  |  |  |
| *Tert*-butyl methyl ether |  |  |  |  |  |  |  |
| *CDCl3* |  |  |  |  |  |  |  |

**Disposal of Waste**

1. No waste from lab should be poured into the sink. Please dispose of waste in appropriately labeled waste disposal containers provided in the waste hood. There will be a separate waste container (“Halogenated Waste”) for dichloromethane and deuterochloroform.

**Chemical Hazards:**

Eye protection and proper gloves should be worn at all times during the experiment and the work should be performed in the fume hood. For student groups using the 372 and 395 nm photoreactors, special UV protective goggles should be used. Bromine is corrosive, toxic, and can cause serious irritation or burns to skin, eyes, and lungs, therefore should only be handled in the fume hood with proper lab goggles and gloves. To minimize chances of student contact, the bromine solution is added to a buret by the instructor. Bibenzyl may cause skin irritation. Cyclohexene is a skin and eye irritant, should not be inhaled, and is stored in a well ventilated area. To minimize student exposure, the instructor quenches all of the reactions in a fume hood. Dichloromethane can cause skin and eye irritation and is a suspected carcinogen and in 2024, the EPA announced new requirements for CH2Cl2 use in the teaching laboratory. We will be following those guidelines. Deuterochloroform causes skin and respiratory irritation, may cause chemical burns, and is suspected of being a carcinogen. All halogenated waste is disposed of in a waste container labeled “halogenated organic waste” while all other waste is disposed of in a waste container labeled “non-halogenated organic waste.”

1. **Instructions with notes for instructors**

Table S2. Completed Table of Physical Properties

| Substance | Formula | MM (g/mol) | Density  (g/ml) | M.P.  (°C) | B.P.  (°C) | CAS # |
| --- | --- | --- | --- | --- | --- | --- |
| Bromine | Br2 | 159.81 | 3.1 | -7.2 | 58.8 | 7726-95-6 |
| Dichloromethane | CH2Cl2 | 84.93 | 1.33 | -96.7 | 39.6 | 75-09-2 |
| 1,2-diphenylethane | C14H14 | 182.27 | 0.978 | 52.0 | 284 | 103-29-7 |
| Cyclohexene | C6H10 | 82.143 | 0.811 | -103 | 83 | 110-83-8 |
| *Tert*-butyl methyl ether | C5H12O | 88.15 | 0.740 | -109 | 55 | 1634-04-4 |
| Deuterochloroform | CDCl3 | 120.38 | 1.50 | -64 | 60.9 | 865-49-6 |

**Additional Spectra**

Figure S3. 1H-NMR spectrum of meso-stilbene dibromide


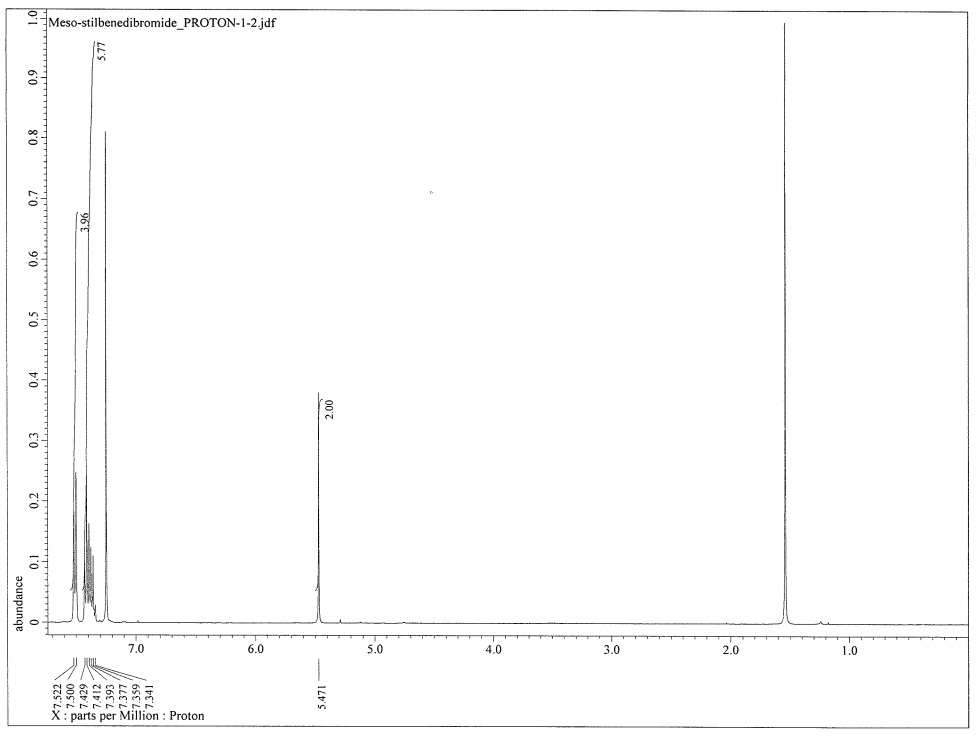


1H-NMR (400 MHz, CDCl3):  7.51 (m, 4H), 7.36 (m, 6H), 5.47 (s, 2H) ppm.

H2O peak at 1.56 ppm and CHCl3 peak at 7.25 ppm.

Figure S4. IR spectrum of meso-stilbene dibromide


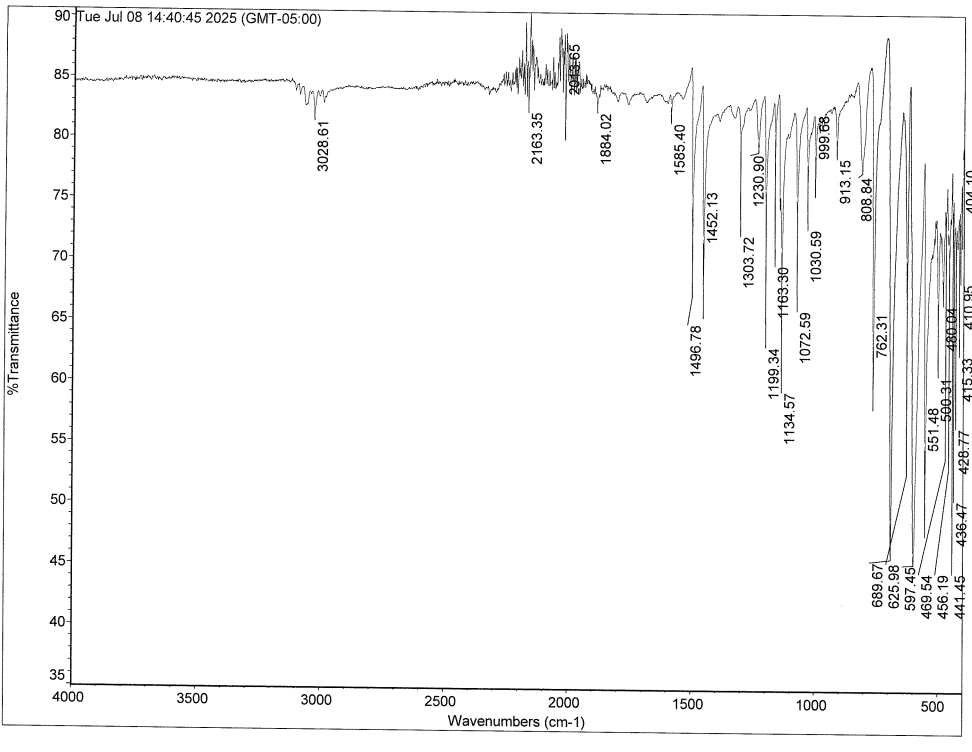


**LED Bromination Lab Worksheet**

Name_________________________________________ Section 01 02 03 04 05

Score:____/30

1. Above is the reaction scheme using liquid bromine to perform this transformation. Calculate the Atom Economy for the above reaction. Note: You need to balance the above equation!
2. Using the following equation, calculate the longest wavelength of light required to break a Br—Br bond given that the bond dissociation enthalpy for the Br — Br bond is 194 kJ/mol. Make sure that your units match! E = Photon Energy, *v* = photon frequency.

NA = Avogadro’s Number = 6.022 x 1023 mol-1

h = Plack’s Constant = 6.626 x 10-34J*s

c = Speed of Light = 2.998 x 108 m/s

What wavelength of light corresponds to your answer?

1. Use the absorption spectrum for Br2 in CH2Cl2 to think of a testable hypothesis that relates max and reaction time for each LED.

At which wavelength would you hypothesize that the reaction works the fastest? What color would this correlate to?

1. Calculate your percent yield of meso-stilbene dibromide. Show all work. Report your melting point range for the purified product. Did it match the literature value of 241 °C? Why or why not.
2. Describe the color and state of your purified product.
3. Using the IR spectrum you attained in class, what evidence do you see for the formation of the product?
4. Using the 1H-NMR spectrum you attained in class, what evidence do you see for the formation of the product?
5. Using all your classmate’s data regarding LED light and reaction time, create a graph of wavelength vs. reaction time. Was your hypothesis from week one confirmed or denied? Why? Paste the graph below.
6. The following graph is the Fluorescence spectrum for a “Violet” LED, purchased from Amazon, that is **different** than our violet photoreactor, but gives off similar intensity. Draw a hypothesis as to whether the reaction would follow the trend in reaction time.
7. Is there anything interesting about the graph obtained from the Violet LED?
8. The following graph is the Fluorescence spectrum for a Pink LED purchased from Amazon.

Do you hypothesize that the reaction occurs faster or slower than with the blue photoreactor? Why?

1. The following Fluorescence spectra were obtained from various sources containing LEDs. Use them to answer the following…

What similarities do all of the graphs share?

1. This reaction was performed in previous years with a 250 W incandescent bulb with the following Fluorescence spectrum.

Describe the graph in as much detail as possible, relating it to the lab you just finished.

The following questions relate to the cost to perform these reactions with various light sources.

**Given**: Average cost of electricity in Illinois currently is 11.47 ¢ / kWh.

**Given**: The Incandescent Bulb used in previous years performed the reaction in 20 min. and pulled 239 W.

**Given**: Each LED light bulb used in this experiment pulls 15 W.

Calculate the experiment cost based on the reaction time for each different LED light source.

Example:

Incandescent bulb:

Therefore, it almost costs 1 penny for each reaction using the incandescent bulb.

Compared to the incandescent bulb, how much cheaper is it to perform the reaction with each specific LED? Use your classmate’s data.

734 nm:

633 nm:

593 nm:

535 nm:

509 nm:

452 nm:

425 nm:

395 nm:

372 nm:
